# “My Kidney Disease, My World as an Arena:” Unpacking the Situation of Adolescents from the Perspective of Postmodern Grounded Theory

**DOI:** 10.17533/udea.iee.v42n3e04

**Published:** 2024-10-12

**Authors:** Keidis Sulay Ruidiaz Gómez, Jasmín V. Cacante Caballero

**Affiliations:** 1 . Nurse, Ph.D. Professor, Programa de Enfermería at Universidad del Sinú EBZ-Cartagena, Colombia: Email: keydis.ruydiaz@unisinu.edu.co. Corresponding author Universidad del Sinú Programa de Enfermería Universidad del Sinú Cartagena Colombia keydis.ruydiaz@unisinu.edu.co; 2 . Nurse, Ph.D. Professor, Facultad de Enfermería at Universidad de Antioquia, Medellín, Colombia. Email: jasmin.cacante@udea.edu.co Universidad de Antioquia Facultad de Enfermería Universidad de Antioquia Medellín Colombia jasmin.cacante@udea.edu.co

**Keywords:** qualitative analysis, quality of life, nursing, grounded theory, adolescent health., análisis cualitativo, calidad de vida, enfermería, teoría fundamentada, salud del adolescente, análise qualitativa, qualidade de vida, Enfermagem, teoria fundamentada, saúde do adolescente

## Abstract

**Objective.:**

To explore the meanings of quality of life for adolescents with chronic kidney disease (CKD).

**Methods.:**

This qualitative study was conducted using a grounded theory situational analysis approach, following the interpretive turn. Four in-depth interviews were conducted with adolescents with CKD, five with parents, and four with healthcare professionals (three nurses and one physician). The collected data were analyzed using situational maps, social world/arenas maps, and positional maps, as proposed by Adele Clarke.

**Results.:**

The characterization of these adolescents’ situations shows that they are the main actors and modify their social role when they suffer from CKD. It is the mothers that traditionally care for them, until they regain their health. The social world map shows the interactions among the worlds of individuals, their families, and the healthcare system, constituting a well-being arena which defines the quality of life for adolescents with CKD. Discursive positions constitute a key element in the discussion concerning the relational dimensions of well-being and the feelings emerging in relation to the disease.

**Conclusion.:**

For adolescents with CKD, quality of life is defined as the state of well-being emerging from the recognition of their own environment in micro-, meso-, and macro-systems, which bring together structural (political, cultural, symbolic) elements, discursive constructions, and the integration of interactions in the social arenas, as well as the representation of the main discourses and their positions.

## Resumen

**Objetivo.:**

Explorar los significados de calidad de vida para el adolescente con enfermedad renal crónica.

**Métodos.:**

Estudio cualitativo con enfoque de teoría fundamentada con análisis situacional de giro interpretativo. Se entrevistó en profundidad a cuatro adolescentes con enfermedad renal crónica, cinco padres de adolescentes y cuatro profesionales de la salud, tres enfermeros y un médico. El análisis de los datos se realizó bajo la construcción de cartografías situacionales, de mundos y arenas sociales y mapa de posiciones propuesto por Adele Clarke.

**Resultados.:**

La caracterización de la situación del adolescente muestra que él es el actor principal que modifica su rol social al padecer la enfermedad renal crónica; la madre asume el cuidado del adolescente hasta que alcance el estado óptimo de salud. El mapa de mundo social muestra las interacciones entre los mundos individual, familiar y de los sistemas de salud en una arena de bienestar que para el adolescente con ERC define su calidad de vida. Las posiciones discusivas denotan el eje de debate para las dimensiones relacionales de bienestar y sentimientos emergentes de la enfermedad.

**Conclusión.:**

Para el adolescente con ERC la calidad de vida se define como el estado de bienestar que surge desde el reconocimiento de su propio entorno en un micro, meso y macrosistema que vincula los elementos estructurales (políticos, culturales, simbólicos), las construcciones discursivas y la integración de las interacciones en las arenas sociales y la representación de los principales discursos y sus posiciones.

## Resumo

**Objetivo.:**

Explorar os significados da qualidade de vida para adolescentes com doença renal crônica.

**Métodos.:**

Estudo qualitativo com abordagem de teoria fundamentada com análise situacional de giro interpretativo. Foram entrevistados em profundidade quatro adolescentes com doença renal crônica, cinco pais de adolescentes e quatro profissionais de saúde, três enfermeiros e um médico. A análise dos dados foi realizada sob a construção de cartografias situacionais, de mundos e arenas sociais e de um mapa de posicionamentos proposto por Adele Clarke.

**Resultados.:**

A caracterização da situação do adolescente mostra que ele é o principal ator que modifica seu papel social ao sofrer de doença renal crônica; A mãe cuida do adolescente até que ele alcance a saúde ideal. O mapa do mundo social mostra as interações entre os mundos do indivíduo, da família e do sistema de saúde em uma arena de bem-estar que para o adolescente com DRC define sua qualidade de vida. As posições de discussão denotam o eixo de debate para as dimensões relacionais do bem-estar e dos sentimentos emergentes da doença.

**Conclusão.:**

Para os adolescentes com DRC, qualidade de vida é definida como o estado de bem-estar que surge a partir do reconhecimento do próprio ambiente em um micro, meso e macrossistema que articula elementos estruturais (políticos, culturais, simbólicos), construções discursivas e integração de interações nas arenas sociais e a representação dos principais discursos e seus posicionamentos.

## Introduction

Adolescence is a stage that begins with pubertal changes, characterized by deep biological, psychological, and social transformations. Many of these generate crises, conflict, and contradictions. The World Health Organization (WHO) indicates that adolescence is the stage that takes place from ages ten to nineteen, with two distinct phases: early adolescence (ages ten to fourteen) and late adolescence (ages fifteen to nineteen).^1^ When chronic kidney disease (CKD) occurs at this stage, it causes changes in everyday life with an impact on daily activities. These are the result of constant clinical controls and analyses, recurring hospital stays, a diet with limited consumption of food and beverages, drug intake several times a day, and frequent invasive procedures which lead to the interruption of school activities.^2^

Treatment of CKD causes medical dependence in adolescents, which interferes with regular activities during this stage, such as playing, studying, developing, and growing.^3^ Furthermore, it affects their own body image, sexual identity, social relations, emotional attachment, and education/vocation. It also results in an adjustment of society’s expectations regarding the level of maturity in their behavior and the values they should have internalized by then if they are to become adults.^4^ Additionally, this condition results in adolescents being separated from their group of friends, increased dependence on their parents or caregivers, and uncertainty resulting from delays in receiving transplants. All the elements mentioned above produce a decline in the quality of life of those suffering from CKD, thus limiting typical development in adolescents.^5^ As a process, this disease causes changes in family routines and social coexistence among adolescents, as they face specific needs in terms of diet and treatment, to name but two, which trigger emotional, physical, and social imbalances. These influence each individual adolescent’s perception of quality of life and their overall sense of well-being.

With all the above in mind, this study is set to examine the situation of adolescents with CKD from the perspective of the meanings of quality of life, based on the situational analysis (SA) model as advanced by Adele Clarke.^6^ The ultimate goal is to present a postmodern perspective on the social complexities and multiplicities of a phenomenon that mobilizes political platforms. Furthermore, through this type of studies, it is possible to assess health as a measure of well-being to be used in the professional practice of nursing. This study provides feedback on comprehensive care incorporating subjective elements that articulate the philosophy of nursing and the acknowledgment of caregiving. In sum, the purpose of this study was to explore the meanings of quality of life for adolescents suffering from CKD.

## Methods

### Design and Participants

This qualitative study was conducted from the perspective of grounded theory with situational analysis (SA) following the interpretive turn as proposed by Adele Clarke^6^. The study was conducted in the city of Cartagena, Colombia, between 2020 and 2022. An inductive-abductive approach was used following the postmodern, poststructuralist, and interpretive paradigms that underpin the understanding of a lived situation, through new conceptualizations that make it different from other epistemological approaches to grounded theory (GT). Clarke draws from Michel Foucault’s work,^7^ specifically his concepts of discourse, discipline, *dispositif* (or apparatus), and conditions of possibility. She also uses the concepts of rhizomes and assemblages, as developed by Deleuze and Guattari.^8^ Based on these metaphors, she proposes *relationalities* between worlds that can be modified and interconnected. She also draws from actor-network theory to discuss relevant nonhuman elements found in a given situation.^6^

The sample of participants was composed of thirteen actors: four adolescents, five parents, and four healthcare professionals (three nurses, one physician). To ensure data variability, participants were included applying the following criteria: adolescents, 10 to 19 years of age;^1^ diagnosed with grade 3 to 5 CKD for over six months; renal replacement therapy or treatment (dialysis, hemodialysis). Parents had to meet the following requirements: parents of an adolescent suffering from CKD, currently cohabitating with them, and having been present throughout the diagnosis and treatment process. For healthcare professionals, the requirements included a degree in internal medicine, nursing, nutrition or psychology, and having provided healthcare at renal units or institutional support services.

Snowball sampling was implemented, followed by theoretical sampling to define the number of participants required to reach theoretical saturation. In this way, the amount of data and information required to create the different maps was delimited.

### Data Collection

Data collection took place by means of in-depth interviews conducted through videocalls or via Google Meet. Adolescents were to be accompanied by their parents or main caregiver. Participants were interviewed while at their place of residence. Interviews were conducted by the project’s main researcher-a nurse and PhD candidate in nursing-from April 2020 to July 2021. Two interview sessions took place, lasting from 30 to 60 minutes. Interview topics included everyday aspects of the adolescent’s life, their diagnosis, changes in their habits since the diagnosis, response to the treatment, and a description of pleasant aspects of their current lives. During the sessions, only one participant refused to continue with the interview due to a complication in their symptoms. Another technique used here was a focus group composed of healthcare professionals and administrators at healthcare institutions (two psychologists, two nurses, one physician). The question leading the conversation was *How does the administrative process of healthcare and treatment of adolescents with chronic kidney disease work?* Each data collection technique was complemented with memos, research matrices, recordings, field notes, and analytical texts.

**Figure 1:**
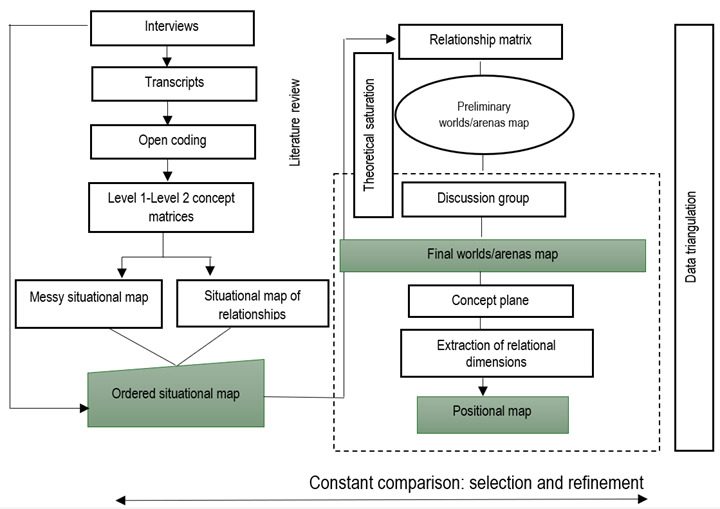
Data Analysis Model based on Situational Analysis GT, as proposed by Adele Clarke

### Quality Criteria

This study complies with criteria of rigor, credibility, reliability, and applicability in its representation of data sources, technical validation, feedback to participants concerning the findings, scientific experience, and assessment. Furthermore, four participants reviewed the extracted concepts and provided feedback concerning the findings.^11^ The lead author carefully considered her own thoughts and experiences to make decision-making criteria explicit, thus reducing risks of bias during data collection and analysis.

### Ethical Considerations

This study complies with the ethical considerations presented in Resolution 008430 issued in 1993 by the Colombian Ministry of Health to regulate healthcare research. It also follows Ezequiel Emmanuel’s seven ethical principles.^12^ Approval was obtained from the Ethics Committee at the Faculty of Nursing, Universidad de Antioquia, Colombia (Minute CEI-FE 2020-03) to apply the informed consent form, record interview sessions, and keep the data secure and confidential. Parents, healthcare professionals, and adolescents gave their informed consent via online forms. They received information about the nature of the study and recorded their authorization. A copy of the consent form was sent to their home for verification purposes.

### Findings

Table 1 shows in detail the ordered situational map, identifying the main elements in the situation of adolescents with CKD, such as main actors, political, sociocultural, symbolic, and environmental elements, as well as discursive constructions.

Actors in the situation. Actors in this situation became main actors; their function was to provide support and face the disease after it has been diagnosed. The main actors in this situation are the adolescents suffering from the disease and experiencing the symptoms and the treatment and modifying their social role. Mothers also serve as main actors, as they assume the role and practices of caregivers so that the adolescents can regain their health and to prevent disease-related complications. Fathers appear as male role models, the providers who respond to basic family needs. When an adolescent is diagnosed with CKD, family ties grow stronger; as a result, peers become allies who share leisure time. Nonhuman aspects constitute another relevant element in this situation: adolescents identify cyclers as nonliving elements that become a part of their everyday lives and relationships, thus establishing networks of social interaction that carry meanings with them.

Political, sociocultural/symbolic, temporal, and spatial elements. Several political or economic elements derived from the healthcare system determine the situation of an adolescent with CKD. Among them, we may mention the increase in medical expenses; access to health services; constantly traveling to other areas to obtain treatment; complying with transplant guidelines and other administrative procedures from the perspective of healthcare providers; and fragmentation in the delivery of healthcare. Culturally and symbolically, body image, sexuality and spirituality are elements that condition this situation. Temporal elements include events in the adolescents’ everyday lives they need to face, such as changes in treatment modalities and the interruption of school activities to undergo the recovery process. The most important spaces for their quality of life are the hospital, renal units, intensive care units, school, and their own home. 

Discursive constructions of individual/collective human and nonhuman actors. Collective actors such as teachers, family and healthcare professionals produced discourses that contributed to an understanding of quality of life. They demonstrated their concern for such issues as adaptation and reaction to the diagnosis; everyday life; the interruption of school activities due to warning signs, symptoms, and complications resulting from the disease; and type of treatment. Discourses concerning nonhuman actors highlighted how adolescents modify their role in life, adjusting to their treatment and complications resulting from their disease. Finally, transplants are the preferred treatment option, but long administrative procedures usually result in failure to obtain one.

Debates and related discourses. The discourses by the participants revolve around topics such as failure to provide adequate care; limited healthcare access; kidney transplants as a life-changing option; age and type of treatment received; loss of interest in typical adolescent activities; rapid changes in the lives of adolescents and their families; development of autonomy versus non-maleficence on the part of healthcare professionals; and intervention opportunities (comprehensive group support). These are some of the most common situations discussed in the interactions among the actors in this context. Through these discourses, it is possible to see how such a complex system is created and reveal emotional aspects, experiences, and changes taking place when someone suffers from a kidney disease. As a result, solutions and intervention alternatives can be offered as part of a micro-, meso-, and macrosystem (Table 1).

**Table 1:** Situational Map for Adolescents with CKD

Individual human elements/actors	Nonhuman elements/actors
Adolescents	Technology - cellphones
Mothers	Cycler
Nurses	Pharmacotherapy
Pediatric nephrologist	Pharmacological intervention
Siblings - Friends - Cousins	Animals
Researcher	Toys
Psychologists - Social workers	Medical devices (access)
Father	Alternative care
Collective human elements/actors	Implicated/silent actors/actants
Teachers	Father
Healthcare professionals	Friends
Family	
Hospitals - Renal Units	
Intensive Care Units	
School - Educational institution	
Transplant unit	
Home	
National government - Healthcare policy	
Public and private healthcare institutions	
Discursive constructions of individual and/or collective human actors	Discursive constructions of nonhuman actors
Adjusting to the disease	Transferring one’s life experience to play
Adolescent autonomy - responsibility	Treatment device
Interrupting academic life	Expectations concerning treatment options
Everyday life / activities	Dietary changes
Absence of relationships with friends	Waiting periods in healthcare
Physical complications due to CKD	Kidney transplant guidelines
Individual reaction to a CKD diagnosis	
Parent suffering	
Body changes	
Age-dependent treatment	
Political/economic elements	Sociocultural/symbolic elements
Increase in medical expenses	Body image
Access to healthcare services	Sexuality
Constant travelling to other areas	Spirituality
Transplant guidelines	Mother empowerment concerning healthcare
Access and opportunity in healthcare services and support from interdisciplinary team	Fear of death
Administrative process (admission, discharge, healthcare provision)	Changing roles (family dynamics)
Healthcare fragmentation	Social life (friends, family, social circle)
	Job opportunities
Temporal elements	Spatial elements
The future: projects and limitations	Hospital
Treatment modality	Renal units
Changes in one’s surroundings, Covid-19 Pandemic	Intensive care units
Interrupting school activities	Social reintegration
Life before vs. after the disease	School
	Home
Major issues/debates (usually contested)	Related discourses (historic, narrative, and/or visual)
Shortcomings in health provision	Positive attitude in face of the disease
Limited healthcare access opportunities	Selective friendships
Kidney transplant as a life-changing option	Changing roles (changes in family dynamics)
Age and type of treatment	Coming to terms with a CKD diagnosis
Lack of interest in traditional adolescent activities	Father as an agent in healthcare
Rapid changes in the lives of adolescents and their families	Overprotective parents
Autonomy vs. non-maleficence in healthcare	
Intervention opportunity - comprehensive group support	

“My social world, my world as an arena.” Adolescent interactions. In the social arena, In the social arena, three worlds interact: the individual world, the family world, and the healthcare system world.

**Figure 2:**
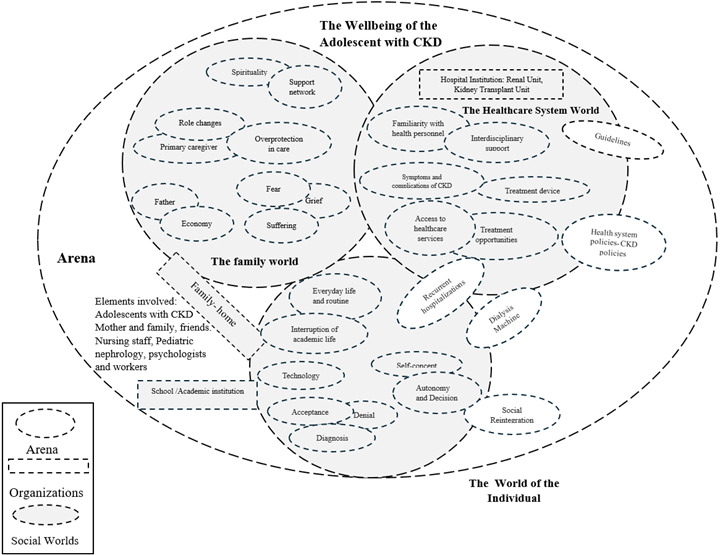
Social world/arenas map of adolescents with CKD

Individual world. Elements represented in this map include the individual’s self-concept, autonomy, and decision-making process. Other elements such as technology, the dialysis machine, recurring hospitalizations, and the interruption of school life condition the way in which adolescents participate in their own treatment and their social reincorporation into an everyday life environment. For adolescents with CKD, their self-concept includes feeling well about themselves and about the people surrounding them; it also includes being satisfied with the way their life is going, their environment, and their behavior, which are interwoven with their interpersonal relationships, job opportunities, academic life, and building a love/family life. To that effect, one of them said: *… my hair and my personality are tamer than before; I used to be rebellious. In the future, I see myself with a transplant, with a career, a family, and children, my family… all together* (Teenager 2, 14 y/o, Female). Because of her own sense of autonomy she can rationalize the complexity of her disease, taking on responsibilities concerning her own care which contribute to her recovery and rehabilitation. Nevertheless, in the context of a chronic disease, there are tensions between dependence and independence. As a result, developing autonomy takes longer than usual and dependence on caregiving from parents increases. About this issue, one of them said: *I go to my grandma’s place, by myself. If I have to go to the unit for a test or a physical, I go alone, and so on* […] *Also, let’s say, if I have to take a pill, or go to dialysis, I mean everything, as a daily routine. If I have to go out, I go out; if there’s something I need to do about my treatment, I do it* (Teenager 2, 19 y/o, Female).

Family world. The family world of adolescents with CKD includes their main caregivers, their tasks, changing roles, feelings, fears, support networks and anything else providing support during the disease. It is usually the mother who becomes the main caregiver, sometimes even being forced to stop working to provide more care and protect the home environment/hygiene: *Moms become empowered when taking care of their kids. They are very careful when it comes to disinfection. They are always paying attention to their emotions. Most mothers are very supportive when it comes to caregiving. They are always very calm, but also very careful* (Health professional 2, female, nurse). Mothers, as the main caregivers, usually adopt an altruistic and tenacious disposition when they need to learn anything concerning the wellbeing of their children. In addition to this, there are also some values that are traditionally associated with the nature of the relationship between mothers and children, which have to do with selflessness and greater affection when compared to the more practical role of fathers as providers: *ever since dialysis began, my husband has always been with us, always ready for any process related to the transplant; he is the one who goes to the HMO, you know how complicated everything is with them* (Mother, 44 y/o). Concerning emotions, adolescents and their families adopt coping strategies to overcome negative feelings. Here, support from health professionals, psychologists, and social workers helps in rationalizing the disease, which contributes to a better treatment and greater patient wellbeing at every level of human life (social, individual, cultural, etc.). One of them explained: *At the kidney unit, psychologists have helped her a lot: they talk to her, they tell her she can live a normal life, there is no reason this should affect her, and she’s taken really well to that* (Mother 4, 52 y/o). In sum, families and adolescents engage in processes that aim to modify, alleviate, strengthen, and transform both their own subjective experiences and the way in which they express their emotions, be them positive or negative. At this point, spirituality and support networks provide strategies to address any problem affecting their emotions.

Health care system world. In the world of healthcare systems, a macro-contextual conception is represented. Elements here include policies regulating health institutions and treatment options, as well as access to treatment and health services. In the arena of adolescents with CKD, access to health services and treatment opportunities contribute to comprehensive healthcare and wellbeing concerning education, information, health promotion, diagnosis, treatment, and rehabilitation, in accordance with

 the principles of quality, efficiency, and opportunity established in Law 100, 1993, which regulates healthcare in Colombia. In the Colombian healthcare system, there are sectoral gaps and obstacles to access treatment and services offered by Health Maintenance Organizations (HMOs), such as referrals, tests, and access to specialists (nephrologists). Nevertheless, there are also health access opportunities, such as the possibility to choose between treatment options, which contributes to a sense of comfort and well-being and thus improves quality of life of adolescents, and, consequently, their families. One of them reported: *The chief said: ‘if you want, we can give you the machine; then they brought the dialysis machine. Every night I get connected; it’s been four years now* (Teeneger 1, 19 y/o, Female). When it comes to adolescents, the healthcare system world shows how healthcare policies and guidelines for attention enforced by health institutions have an impact on healthcare opportunities and access. Adolescents and their families are immersed in this world as citizens in a welfare state that offers healthcare protections and considers healthcare to be a human right. In this context, health care constitutes a relational experience between the individual and state organizations from a perspective of power and resistance. As a result, intersubjective meanings about well-being and the way society works are constructed.

**Figure 3:**
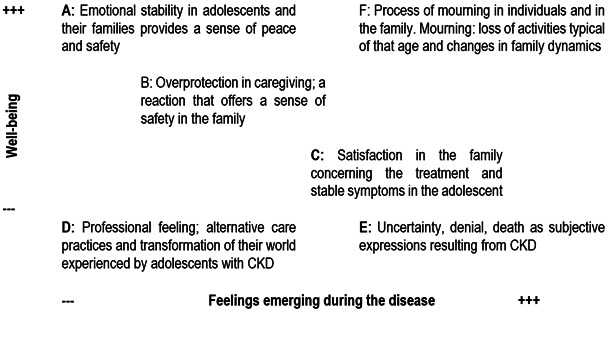
Positional map 1: Well-being - feelings emerging during the disease

Positional map: discursive positions of actors in the situation. The wellbeing of adolescents with CKD is a shared interest among the adolescents themselves, the medical team, and the family. Well-being in this context is understood as the subjective state reached by adolescents with CKD when there is a balance between their health and the social, psychological, cultural, economic, and political dimensions in their lives. When such balance is achieved, they can form positive judgments concerning their own reality. In this sense, the relationship between this shared interest and emerging feelings point to a great level of well-being in the discourses coming from different actors surrounding adolescents with CKD; as a result, they also reach a state of balance and peace. Along its vertical axis, the positional map represents what quality of life (well-being) means to adolescents; the horizontal axis represents a major debate issue: the feelings emerging during the disease. On the other hand, each discursive key was organized following several representative positions in a scale from greater (+) to lesser (-), as follows in Figure 3:

## Discussion

This situational analysis of adolescents with chronic kidney disease shows that the meanings of quality of life they construct are the result of an interpretation of their feeling of well-being during the disease. Such interpretation emerges from their self-concept, the exercise of their autonomy, and support from their families and society, specifically healthcare institutions and the way they handle their treatment in adolescents’ individual world, their self-concepts allow them to shape their personalities in order to bond and engage in social competition with peers. As a result, they develop their self-confidence, learn how to overcome obstacles, and reach their current and future goals.^13^ Through a constant search for life’s meaning, adolescents can strengthen their analytical and critical skills and develop a stronger identity, reinforcing their values, ideas, and connections. 

Consequently, committing to positive thinking when facing disease results in greater control over situations (autonomy);^14^ experiencing positive emotions provides resources to successfully face health problems, resulting in healthier feelings such as joy, love, satisfaction, and hope.^15^ Thus, when adolescents and their families develop positive emotions, they are protecting themselves against the stressful or traumatic situations they are going through and learn how to cope with stressful events.^15^ At the family level, adolescents construct a system of interactions which change the structure of the family and have a social impact. As a result, social changes brought about by postmodernity-such as the deinstitutionalization of family; changing conceptions of legitimate or illegitimate families; divorce; assisted fertilization; changing social representations of sexual diversity and gender; parenthood; and even the role of women in the family and in society-alter family functions and overload family roles.^16^ In the context of overloads and the quality of life of caregivers for children with cancer, it has been suggested that said caregivers-in addition to coping with the diagnosis, mood changes in their relatives, uncertainty concerning the disease, and attending to other needs-should be concerned for their own emotional conflicts, e.g., abandoning or postponing their work or social activities, economic problems, or neglecting their own health, which modifies their daily life dynamics and is related to their spirituality.^17^

In fact, spirituality offers some solace to adolescents and their families as they seek for meaning in the challenging times they are going through as a result of CKD. Furthermore, it offers self-control, emotional comfort, and physical and mental balance that can be lifechanging. Balboni *et al.*^18^ present spirituality as a subjective form of knowledge, which should be seen from a broader perspective. When going through situations of disease, beyond the evident challenges, there is also room for personal growth, gaining a new perspective on life and finding a chance to help others. 

Another issue to be considered is the way in which the Colombian healthcare system has introduced changes in the way it deals with chronic diseases. According to the Pan American Health Organization,^19^ healthcare systems should provide access to high quality equipment and guidelines to offer services in a timely, accessible, and fair manner, complying with healthcare policies and ethical mandates. The Colombian Fund for High-Cost Diseases considers CKD as a catastrophic disease, which negatively affects a self-managed system. The model through which the disease is addressed includes several processes such as pathology detection, specific treatments, and service provision at various levels, which ultimately condition clinical practice.^(20^. As a result, access to health services and treatment opportunities (considering that chances to receive a transplant are limited) contribute to achieving a degree of well-being in the arena of adolescents with CKD. As mentioned before, components of well-being in this respect include health education, information, promotion, diagnosis, treatment, and rehabilitation, in accordance with the principles of quality, efficiency, and opportunity as established in Law 100.^21^

The relationship between adolescent well-being and the feelings resulting from their diagnosis lead to an exploration of the individual’s subjective feelings, as pointed out by Kogon and Hooper.^22^ Emotions and feelings occurring during the disease emerge as a natural response to the adaptation and rationalization process in the face of a chronic disease diagnosed during adolescence. Furthermore, the feeling of losing control as a result of the diagnosis, the warning signs and symptoms, dependence on caregivers, a decline in physical integrity, and a restricted lifestyle negatively affect the emotional condition of adolescents.

An analysis of the phenomena related to quality of life from the perspective of adolescents and the interaction with their environment results in meaning-construction processes, which provide nursing scholars with a critical, interpretive perspective. Looking into the social events that surround human beings and attributing meaning to them can bring nurses closer to understanding the meaning adolescents give to their disease as a process. This approach can help solve problems in nursing practice and improve the quality of care offered during treatment. This study offers a critical analysis of real phenomena experienced by adolescents with CKD from the perspective of individuals, society, families, and healthcare professionals. A major contribution of this study is the use of a qualitative methodology following a social approach-one which is rarely used by nursing scholars in Colombia-to analyze the complex situation of adolescents from different perspectives. Furthermore, the theoretical, philosophical, and methodological tools used here provided a deeper understanding of the phenomenon being researched. This study adopted an interdisciplinary approach, in which interrelations were studied as social constructions from a perspective that brings together social, cultural, political, economic, and individual spheres. The theoretical approach presented here offers a specific definition of the quality-of-life construct in adolescents with CKD, associating it with the concept of well-being. Consequently, quality of life is understood at a subjective level as a relationship between sensitizing elements which contribute to the careful consideration and interpretation of the meanings that emerge when suffering from a chronic disease.

Some of the limitations of this study include difficulties during the data collection stage, as interviews had to be conducted online due to the restrictions resulting from the Covid-19 pandemic; this may have interfered in the process due to network failures. Furthermore, there may have been some bias resulting from the absence of a field journal to offer a more detailed account of interviewees’ attitudes. Additionally, the distance of online interviews may have prevented interviewers and interviewees from forming stronger bonds. A major obstacle was the typical complications of CKD, which contributed to worsening symptoms in the adolescents and concomitant negative emotions in parents. This resulted in some of them refusing to participate in second interviews, which made it difficult to contrast their data.

In conclusion, looking into the situation of adolescents from the perspective of grounded theory situational analysis allowed us to determine that the meanings of quality of life for adolescents are related to the emotional, physical, economic, and family stability that emerges during the CKD process. The state of well-being results from their acknowledgment of their own environment as part of a micro-, meso-, and macro-system which integrates structural elements (political, cultural, symbolic), discursive constructions, and interactions in social arenas, as well as the representation of the main discourses and positions. In this sense, the shared domains of the state of well-being include their physical health, emotional balance, family support, economic resources, and adolescents’ participation in healthcare services. Major components also include their autonomy, self-concept, body image, and spirituality.
